# ATP-dependent chromatin remodelers in prostate cancer progression and therapeutic resistance

**DOI:** 10.1210/endocr/bqag103

**Published:** 2026-09-16

**Authors:** Lillian M Torres, Clayton Yates, Charlotte L Bevan, Michael R Freeman, Moray J Campbell

**Affiliations:** Department of Urology, Cedars-Sinai Health Sciences University, Los Angeles, CA 90048, USA; Department of Pathology, Johns Hopkins University School of Medicine, Baltimore, MD 21287, USA; Department of Oncology Sidney Kimmel Comprehensive Cancer Center, Johns Hopkins University School of Medicine, Baltimore, MD 21287, USA; Department of Surgery and Cancer, Imperial College London, London, UK; Department of Urology, Cedars-Sinai Health Sciences University, Los Angeles, CA 90048, USA; Department of Biomedical Sciences, Cedars-Sinai Health Sciences University, Los Angeles, CA 90048, USA; Department of Oncology, Karmanos Cancer Institute, Wayne State School of Medicine, Detroit, MI 48201, USA

**Keywords:** ATP-dependent chromatin remodeling, prostate cancer, epigenetic

## Abstract

ATP-dependent chromatin remodelers (ACRs) have emerged as central determinants of prostate cancer (PCa) progression and therapy resistance. Organized into 4 mechanistically distinct families (SWI/SNF, ISWI, CHD, and INO80/SWR), ACRs govern nucleosome positioning genome-wide and thereby occupy a central position in the epigenomic regulatory landscape that dictates where and when transcription factors, including the androgen receptor (AR), can engage chromatin. This review discusses ACR dysregulation in PCa through both mutational and non-mutational mechanisms. These are illustrated by discussing how the functional consequences are highly context-dependent, varying with disease stage, prior treatment exposure, and tumor ancestry. Loss of each of the tumor suppressors RB1, TP53, and PTEN generates specific ACR dependencies that are potentially therapeutically exploitable, including synthetic lethal relationships between PTEN deficiency and SWI/SNF ATPase activity. Across the spectrum of AR signaling states, from hormone-sensitive disease through therapy-resistant neuroendocrine and double-negative PCa subtypes, ACR complex composition and genomic targeting are continuously reprogrammed to enable and sustain lineage plasticity and endocrine therapy escape. Therapeutic strategies targeting SWI/SNF, ISWI, and INO80/SWR complexes are at varying stages of preclinical and clinical development and are attractive novel avenues to target therapy-resistant PCa.

A combination of mutational events and dynamic changes to the cancer epigenome underlie aberrant gene expression. The changes to the epigenome in turn arise from dysregulated chromatin states, of which nucleosome arrangements are a fundamental to establishing chromatin accessibility by transcription factors. Nucleosomes comprise a histone octamer wrapped by ∼147 bp of DNA and their position is mediated by the superfamily of ATP-dependent chromatin remodelers (ACRs) (1-3). Furthermore, other mechanisms that shape chromatin states, including DNA methylation and post-translational histone modifications, are interdependent with ACR function.

Research in epigenetic mechanisms associated with prostate cancer (PCa) has revealed genome-wide differential methylation patterns during progression, including aberrant CpG island hypermethylation at tumor suppressor gene promoters (4, 5). In parallel, the discovery that the hormone nuclear receptors androgen receptor (AR) and estrogen receptor alpha (ERα) function as platforms for the recruitment of histone-modifying complexes placed histone modification interactions at the center of hormone signaling, particularly at oncogenic enhancer networks (6). Research into these altered epigenomic mechanisms was aided by the ability to interrogate these events at single gene loci or genome-wide, using for example, ChIP- or bisulfite-based approaches.

By contrast, examination of nucleosome positioning, which is required to examine ACR functions, presented a more challenging technical barrier. Within the past decade, the development of ATAC-seq (7) has enabled genome-wide assessment of nucleosome positioning. In parallel, large-scale cancer genomic data analyses revealed that ACR subunits are mutated in approximately 20% of cancers, with significant representation in PCa (8). Furthermore, insights on chromatin organization suggest that ACRs can act to guide where and when the DNA methylation and histone modifications can operate (9, 10), and therefore may occupy an upstream, gatekeeping position in the epigenomic hierarchy. The interrogation of these epigenomic interactions, and the orchestrators of these dynamic remodeling steps, represent opportunities to understand nucleosome actions in cancer development. For example, as illustrated in this review, altered ACR events impact nucleosome repositioning in PCa and thereby shape AR access to genomic binding sites in the transition from hormone-sensitive to AR signaling inhibitor (ARSI)-resistant disease. Understanding the impact of ACRs on epigenomic changes that contribute to PCa progression is the focus of the current review.

## The biological framework of ATP-dependent chromatin remodelers

### Discovery in yeast and conservation in humans

Sequence-specific roles were discovered for transcription factors to control gene expression and cell fate were discovered in the 1980s (11). This raised the question of how transcription factors overcome the impediment of condensed nucleosomes. Serendipitously, 2 groups answered this question by undertaking yeast loss-of-function genetic screens in different phenotypic contexts. Dr. David Botstein's group focused on mechanisms that allow yeast to survive in response to switching carbon source from sucrose to glucose, thereby defining the first Sucrose Non-Fermenting (Snf) gene, Snf1 (12-14). Similarly, Dr. Ira Herskowitz's group addressed mating-type switching, and defined the SWItching (SWI) loci corresponding to 5 Swi genes (15). Subsequently, Snf2 and Swi2 were shown to be subunits of the same complex, encoding an ATPase with chromatin remodeling activity (16-19). Collectively, these research groups established that the SWI/SNF complex controlled chromatin structure, which was central to eukaryotic transcription and therefore impacted cell fate choices (reviewed in (20)).

### Four major families of ATP-dependent chromatin remodelers orchestrate accessibility, organization, and surveillance

**Figure 1:**
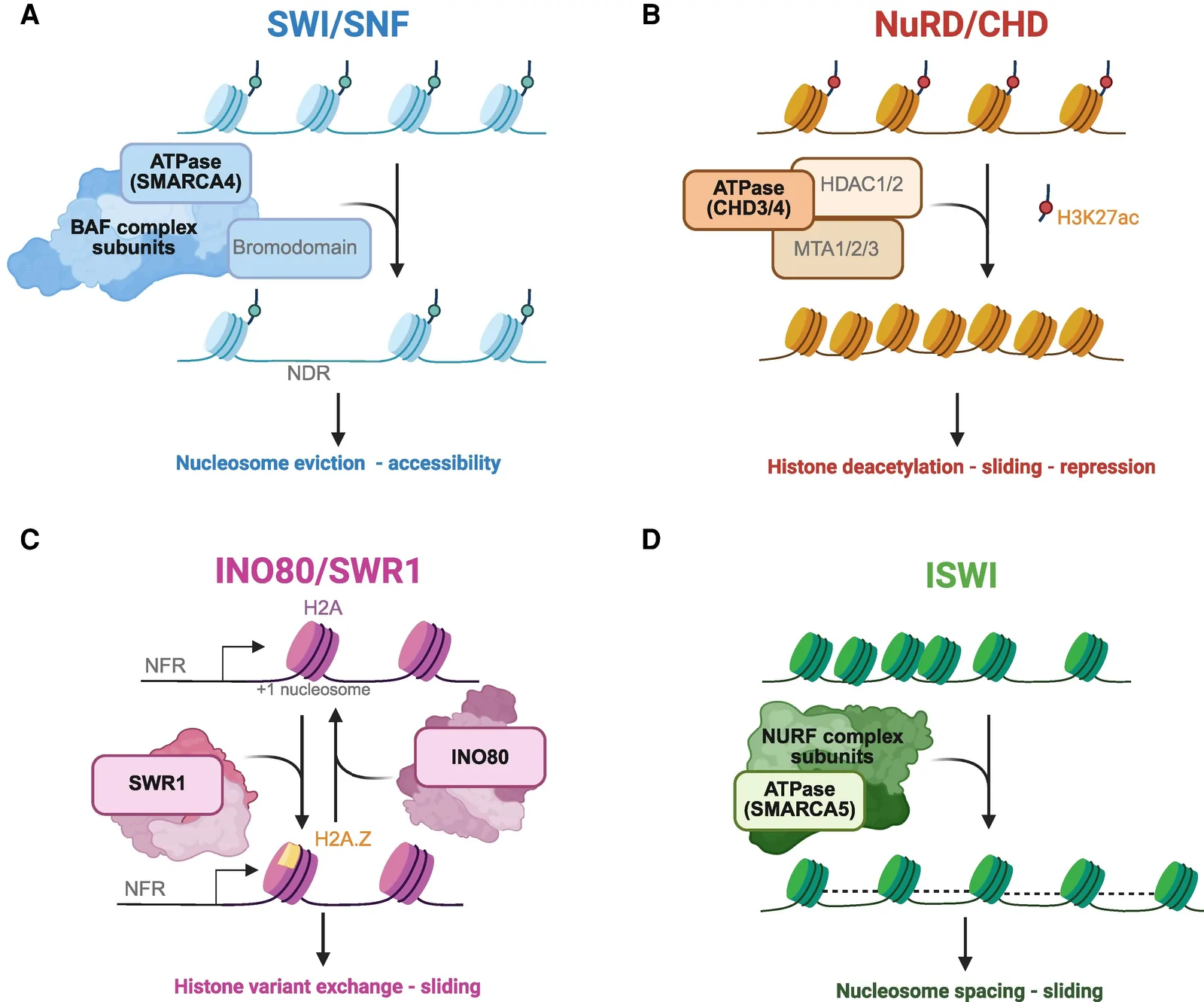
Summary of ATP dependent chromatin remodeler family mechanisms. A) SWI/SNF family is represented by the SMARCA4 ATPase interacting with the BAF Complex to slide or evict nucleosomes promoting chromatin accessibility. B) Chromodomain ATPases such as CHD3 or CHD4 interact with Nucleosome Remodeling and Deacetylase Complexes (NuRD) to remove acetylation marks, illustrated by H3K27ac removal, for example. C) Deposition and removal of a representative histone variant enriched in PCa, H2A.Z, by SWR1 and INO80, respectively. D) Nucleosome sliding and spacing is organized by NURF Complex containing the ISWI ATPase SMARCA5. Created in https://BioRender.com.

SNF2-family members in humans share a conserved catalytic domain to initiate ATP hydrolysis, but diverge extensively in their accessory domains, allowing variation in subunit composition and different biological functions (21). There are more than 30 complexes in human cells governing distinct gene regulatory networks (reviewed in (22)). Based on ATPase domain architecture and mechanistic specialization, the human SNF2 superfamily is organized into 4 major subfamilies: SWI/SNF, ISWI, CHD, and INO80/SWR (23). Each subfamily executes a distinct set of nucleosome transactions, namely eviction, spacing, sliding, and histone variant exchange, respectively (Fig. 1), although there are context-specific exceptions suggesting further complexity to the control of these functions. Collectively, therefore, ACRs can rapidly shape chromatin architecture to establish accessibility and organization, and to undertake surveillance of nucleosomes.

#### Accessibility

There are 3 multi-subunit SWI/SNF BRG1/BRM-associated factor (BAF) complexes in humans. The canonical BAF (BAF), polybromo (p)BAF, and noncanonical (nc)BAF, each contain either SMARCA4 (BRG1) or SMARCA2 (BRM) as the catalytic ATPase. These complexes consist of multiple subunits to eject nucleosomes and create nucleosome free regions (NFRs) that license transcription factor access, for example at lineage-defining enhancers and promoters (24). The SMARC name (SWI/SNF Related, Matrix Associated, Actin Dependent Regulator of Chromatin) designates a gene family based on domain structure and function. The SMARCA subfamily (subfamily A) encode the catalytic ATPases. SMARCA4 (BRG1) and SMARCA2 (BRM) are bona fide SWI/SNF ATPases, while SMARCA5 (SNF2H) and SMARCA1 (SNF2L) are ISWI family ATPases that carry the SMARCA designation. The remaining subfamilies encode structural and regulatory subunits of SWI/SNF complexes. SMARCB1 (SNF5/INI1, subfamily B) is a core BAF subunit and established tumor suppressor; SMARCC1 (BAF155) and SMARCC2 (BAF170) (subfamily C) serve as scaffold subunits of BAF and pBAF; SMARCD1/2/3 (BAF60a/b/c, subfamily D) are regulatory subunits that confer tissue-specific targeting; and SMARCE1 (BAF57, subfamily E) contains an HMG domain implicated in DNA binding and complex targeting.

#### Organization

The human ISWI family uses SMARCA5 (SNF2H) or its paralog SMARCA1 (SNF2L) as the catalytic ATPase. The ISWI complexes, such as Chromatin Remodeling Complex (WICH) and Nucleosome Remodeling Factor (NuRF), function to space nucleosomes evenly. This process establishes and maintains ordered chromatin arrays that contribute to transcriptional activation and precision, heterochromatin organization, and the re-establishment of chromatin architecture following cell division and DNA replication.

The Chromodomain Helicase DNA-binding (CHD) family encompasses 9 human paralog ATPases, CHD1-9, which are distinguished by tandem chromodomains that mediate interaction with methylated histones. The best characterized CHD complex is NuRD (Nucleosome Remodeling and Deacetylase), which contains CHD3, CHD4, or CHD5 as its catalytic subunit and copurifies with histone deacetylase activity, thereby linking nucleosome repositioning to the histone modification landscape.

#### Surveillance

The INOsitol requiring 80 (INO80) and SWi2/snf2-Related 1 (SWR1) complexes are grouped together based on shared structural features that includes a split ATPase domain and the presence of the Rvb1/Rvb2 ATPase subunits. INO80 mediates nucleosome sliding and eviction, particularly at transcription start sites (TSS), as well as participating in repair of DNA damage repair and replication fork stability. The human SWR1 ortholog, SRCAP (SNF2-Related CREBBP Activator Protein), and the related TIP60-p400 complex specialize in histone variant exchange, depositing the histone variant containing dimer H2A.Z/H2B in place of canonical H2A at gene regulatory regions, including the +1 nucleosome position immediately downstream of the TSS. INO80 and SWR1/SRCAP operate as antagonistic systems whereby SWR1/SRCAP deposits H2A.Z and INO80 removes it (25).

### Functional diversity of ACR through combinatorial assembly

**Figure 2:**
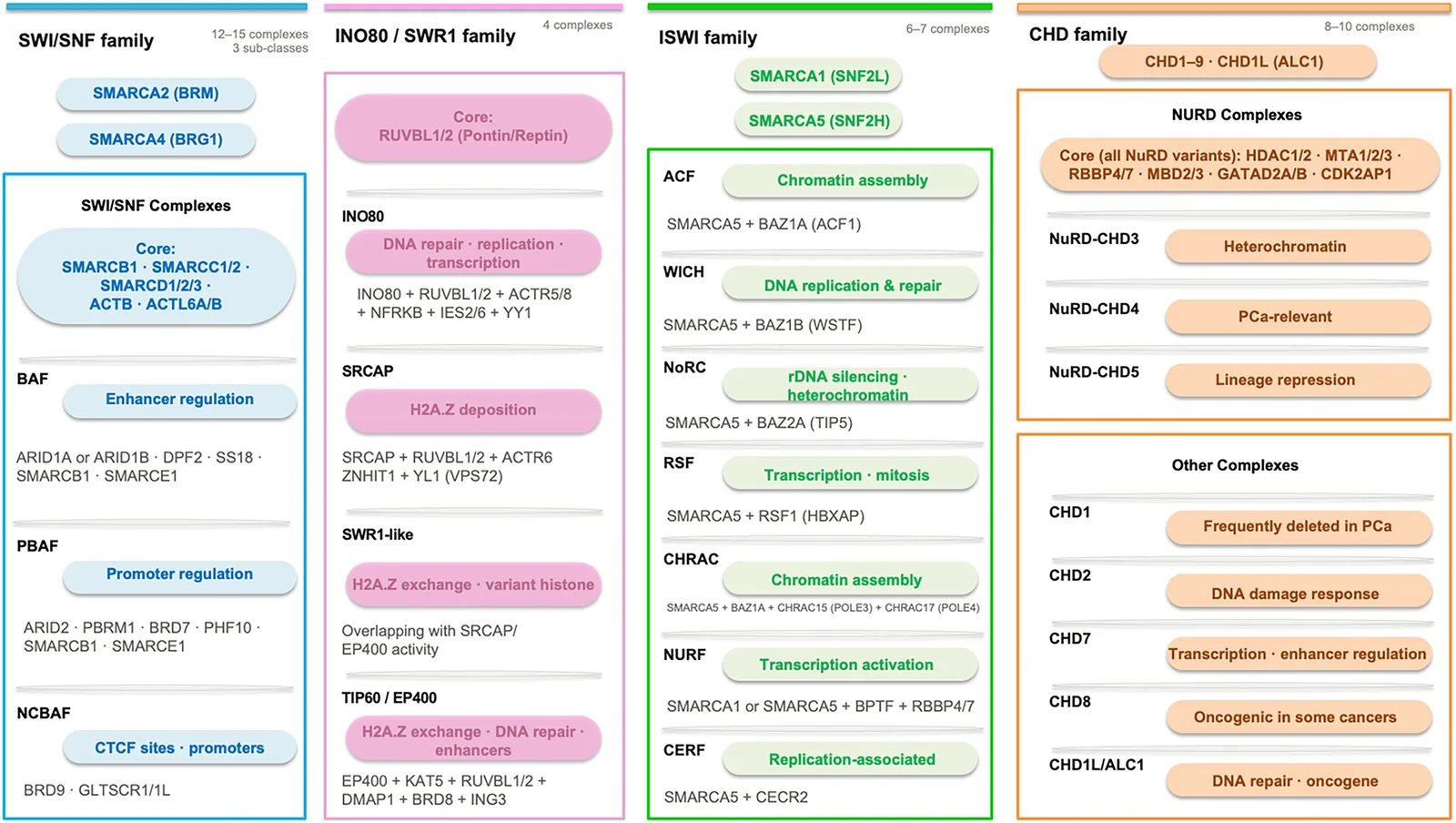
Summary of 4 major ACR families. Complexes and predominant functions or oncogenic relevance are denoted within each complex or subclass.

The combination of ATPases with different accessory subunits generates functional diversity. Substitution of one accessory subunit for a paralog can alter complex targeting, enzymatic activity, and protein–protein interactions without changing the catalytic ATPase. For example, within the SWI/SNF family, exchange of the ARID subunit (ARID1A/B or ARID2) or the SS18/SS18L1 subunit distinguishes BAF, pBAF, and ncBAF complexes, which have nonredundant functions in enhancer regulation and lineage specification (Fig. 2). Similarly, SMARCA5-containing complexes, such as WICH or NoRC (Fig. 2), determine whether SMARCA5 activity is directed toward nucleosome spacing during replication, heterochromatin silencing, or ribosomal gene repression (26).

## ACRs impact prostate cancer progression and therapy responsiveness

### Prostate lineage commitment in normal and cancer biology

PCa occupies an unusual position among solid tumors, as this malignancy is defined more by transcriptional dysregulation (27, 28) compared to mutational burden (29). This suggests that epigenomic events combine with mutational events to drive disease, as evidenced by the fact that expression of chromatin remodelers are commonly altered (30). In addition, lineage plasticity and ARSI resistance can arise in murine models (31, 32) and patients (33) more quickly than predicted by the change in frequency of genomic alterations. Moreover, therapies targeting the epigenome can augment ARSI effectiveness (34, 35).

The switch in transcriptional actions in PCa is illustrated by the pleiotropic functions of the AR. In normal prostate epithelial cells, and even in early-stage disease, the AR cistrome coordinates gene regulatory networks that maintain luminal commitment (36). However, in later disease stages, and in response to ARSI therapeutic restraint, the AR is co-opted to alternative lineage-specific gene regulatory networks in part through the altered pioneer factor activity of FOXA1 (37) and often in cooperation with HOXB13 (38, 39). It is worth noting that the epigenomic architecture that sustains luminal and other lineage programs is largely disassembled in mitosis and reassembled in daughter cells. ACRs play key functions in mechanisms to sustain and restore this epigenomic architecture through mitosis (40). Perhaps reflecting these central roles, the ACR families are recurrently altered, functionally co-opted, and mechanistically central to the transcriptional reprogramming that drives PCa initiation, progression, and resistance to therapy, and this suggests they are not simply permissive or correlative of altered AR activity.

### Chromatin accessibility, organization, and surveillance in PCa

**Figure 3:**
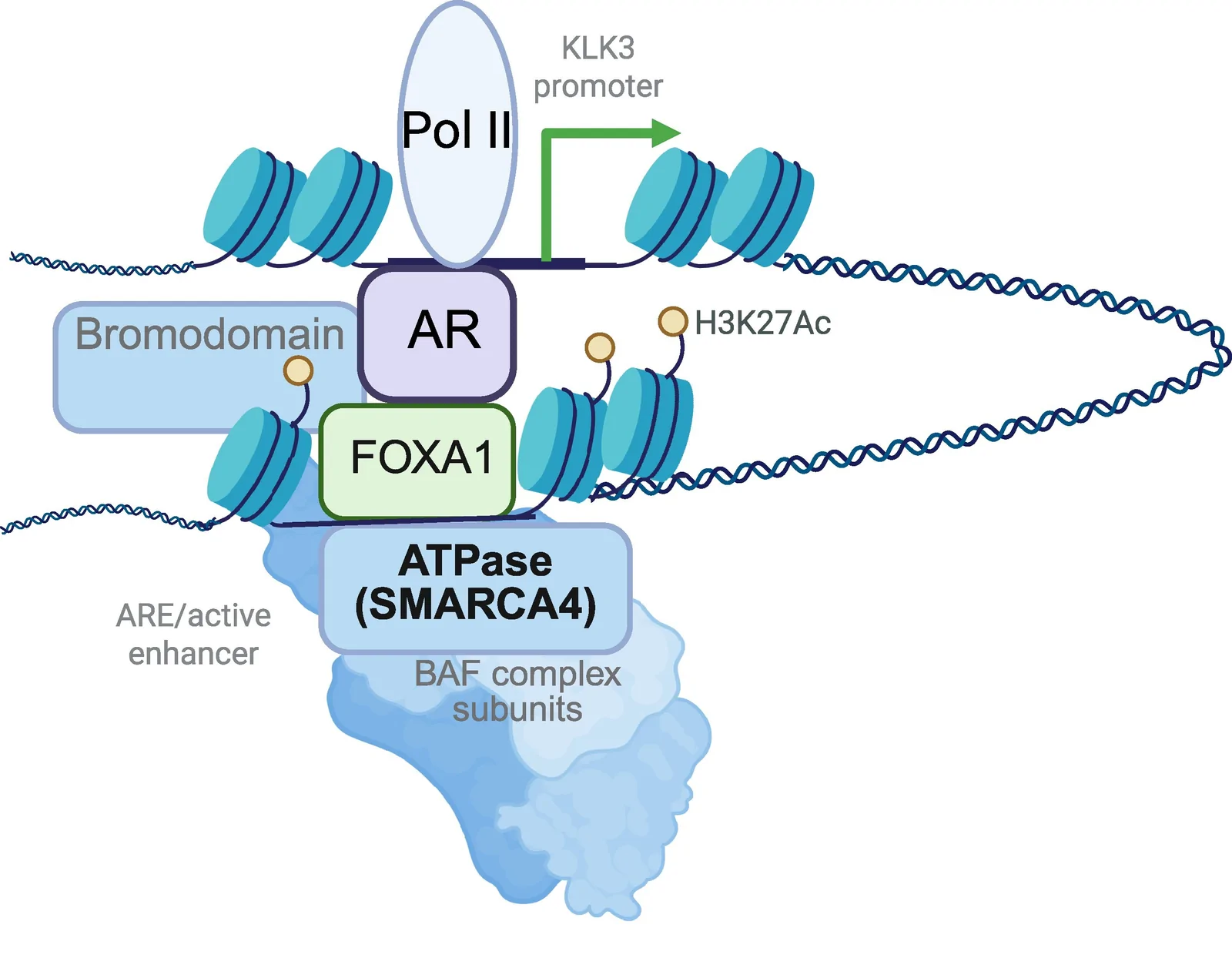
Epigenomic interaction at AR target loci. SMARCA4 remodels chromatin to maintain an open enhancer supported by FOXA1 pioneer activity. Active AR enhancers marked by the androgen response element (ARE) are flanked by H3K27ac-marked nucleosomes. FOXA1 co-binds with AR at enhancers and cooperates to activate luminal gene expression. PolII completes the enhancer-promoter loop to drive KLK3 transcription. Created in https://BioRender.com.

As the orchestrators of nucleosome eviction, SWI/SNF complexes enabling chromatin opening and accessibility. BAF and pBAF complexes are recruited to enhancers through interactions with sequence-specific pioneer factors such as FOXA1, and their remodeling activity is required to sustain (or maintain (41)) nucleosome-depleted regions at AR-bound regulatory elements genome-wide (Fig. 3). Complementing the somatic alterations in ACRs (8) are changes in expression of the various subunits that alter complex stoichiometry and assembly. The ATPases SMARCA4 and SMARCA2, for example, show reciprocal expression changes across PCa progression that alter SWI/SNF complex composition and activity, without acquiring somatic mutations (42, 43). Specifically, SMARCA2 tends to be lost in advanced PCa, while SMARCA4 is more variably expressed (44).

Nucleosome spacing shapes chromatin organization in other regions beyond enhancers, including gene bodies, heterochromatin domains, and replication-associated chromatin. These functions are associated primarily with the ISWI and CHD families. Rather than displacing nucleosomes, ISWI complexes use ATP hydrolysis to slide nucleosomes into evenly spaced positions, creating the ordered chromatin architecture that underlies transcriptional precision, heterochromatin boundary maintenance, and the faithful re-establishment of chromatin structure following DNA replication (45, 46). In PCa, gains and losses of SMARCA5 expression disrupt the normal balance between nucleosome spacing and accessibility, with consequences for both gene body transcription and the fidelity of lineage-specific gene repression. Possibly reflecting these diverse roles, there is varying SMARCA5 expression in PCa (47, 48) with increased expression impacting AR activity (49), and decreased expression selectively impacting the vitamin D receptor in African genomic ancestry PCa (50). The diversity of these functions possibly also reflect contributions of circSMARCA5 operating as a molecular sponge to limit PCa proliferation (51).

Finally, the INO80/SWR family survey genome-wide chromatin structure (52), including at sites of DNA damage (53), replication stress, and transcription-replication conflicts (54). The INO80/SWR family often function cooperatively with specific members of the CHD and ISWI families. INO80 is recruited to sites of DNA double-strand breaks, where it repositions nucleosomes to facilitate the access of repair machinery (55), and its activity is coupled to the exchange of histone variants at damaged loci (56). Chromatin surveillance takes on particular significance in the context of the high replication stress in PCa (57, 58).

The 3 ACR functional modes of nucleosome eviction, organization, and surveillance are not independent, but instead interact continuously to maintain chromatin states that define prostate epithelial identity. Chromatin opening by SWI/SNF at AR enhancers depends on the nucleosome-organized chromatin substrate provided by ISWI complexes in gene bodies and flanking regions. Chromatin surveillance by INO80/SWR at replication-stressed loci influences the histone variant composition that in turn affects SWI/SNF and CHD recruitment. CHD family members, and in particular CHD1 and the NuRD-associated CHD4, couple nucleosome repositioning to histone modifications, linking the physical organization of chromatin to the maintenance of repressive or active epigenetic domains and transcriptional programs (59-61). This functional interdependence means that perturbation of any single remodeling family has the potential to propagate through the chromatin regulatory network in ways that are not predictable from the function of that family in isolation.

## Roles of ACR across the spectrum of AR signaling in PCa progression

Although reviews have comprehensively described context-dependent AR interactors and AR signaling evolution across disease states (62, 63), the ACR dependencies that enable each transition are less comprehensively covered.

### AR expressing hormone-sensitive prostate cancer

Early studies established that FOXA1 anchors the AR cistrome, that this was functionally required for regulation of genes that control luminal differentiation, and that SWI/SNF complexes maintained this accessible chromatin configuration (Fig. 3) (64). It is also clear that changes to SWI/SNF targeting can shift AR-enhancer interactions. Degradation of the SWI/SNF ATPases through AU-15330, a dual degrader targeting SMARCA2/4, collapses cis-regulatory chromatin elements and evicts AR and FOXA1, thereby limiting AR-bound super-enhancers and their long-range looping interactions, and synergizing with enzalutamide to suppress AR-driven transcription in vitro and in xenograft models (64). Complementary pharmacology approaches with the dual SMARCA2/4 ATPase inhibitor FHD-286 similarly reduces FOXA1-mediated AR activity and reduces AR occupancy at tumor-associated AR binding sites, producing selective cytotoxicity in AR-positive cells (65). Likewise, the BAF57/SMARCE1 subunit provides a biochemical tether between AR and SWI/SNF through binding to the AR DNA-binding domain and hinge region and is required for AR residence on chromatin and AR-dependent proliferation (66). Collectively these results reinforce that AR transcriptional output is contingent on intact SWI/SNF activity.

The TMPRSS2-ERG fusion, present in approximately 50% of hormone-sensitive PCa in European ancestry patients, adds a further layer of SWI/SNF dependency in this disease state (67). TMPRSS2-ERG directly binds BAF chromatin remodeling complexes through interaction with the BRD9-containing ncBAF subcomplex and is required for ERG-driven oncogenic transcription and transformation (68). The ancestry-specific prevalence of ETS fusions therefore shapes not only the AR cistrome but may also impact the specific ACR dependencies that impact hormone-sensitive disease.

ISWI complexes also function in AR-dominant PCa. The NURF complex subunit Bromodomain and PHD Finger Transcription Factor (BPTF) enhance chromatin accessibility at AR target sites and stabilizes the AR-FOXA1 interaction. Specifically, BPTF depletion reduces AR occupancy at a subset of enhancers and impairs AR-driven transcription (49). These findings support the concept that not only nucleosome eviction by SWI/SNF but nucleosome spacing and organization by ISWI complexes may function as a prerequisite for the productive AR-FOXA1 axis at shared regulatory elements. The ISWI-mediated chromatin organization and SWI/SNF-promoted chromatin opening are not independent systems but reflect a functional dependency at shared regulatory sites.

**Figure 4:**
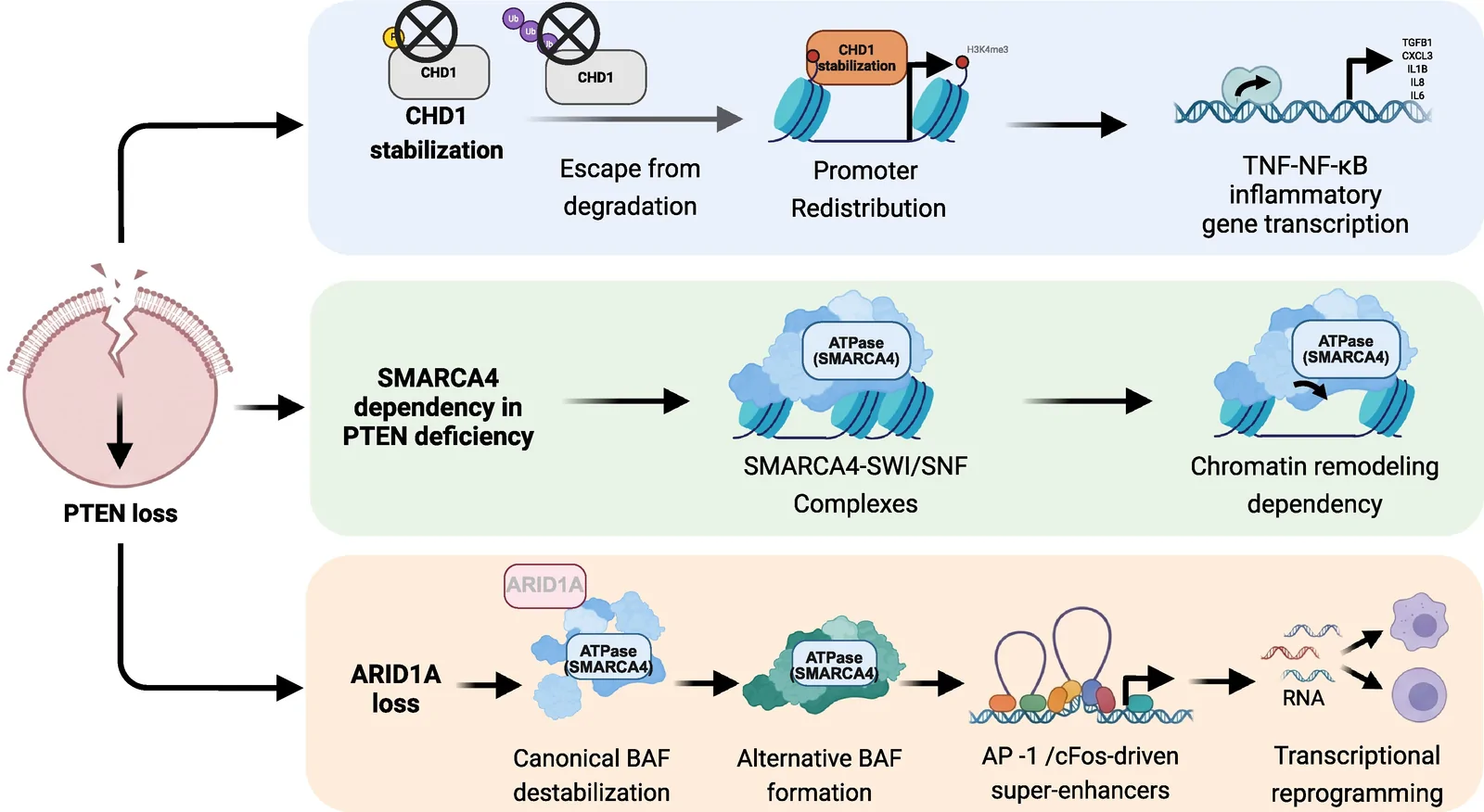
PTEN loss creates ATP-dependent chromatin remodeler dependencies. (1, top) CHD1 is stabilized via escape from PTEN/AKT/GSK3B/B-TrcP inhibition. Its redistribution to H3K4me3-marked promoters promotes chromatin accessibility and transcription of NF-kB inflammatory genes. (2, middle) SMARCA4 becomes essential in PTEN deficiency driving nucleosome eviction and chromatin remodeling through disease progression. (3, bottom) ARID1A guides canonical BAF to chromatin. Following ARID1A loss canonical BAF is destabilized and alternative BAF complexes form enhancing AP-1/cFos-driven transcriptional superenhancers and transcriptional reprogramming. Created in https://BioRender.com.

PTEN loss is common in early-stage and AR-dependent PCa and at higher frequency in ARSI-resistant cancer (30, 69). By activating the phosphoinositide 3-kinase (PI3K)/Akt signaling pathway, PTEN loss reshapes the transcriptional and metabolic landscape in ways that generate specific dependencies on ACRs and possibly create therapeutically exploitable synthetic interactions. The most well-characterized of these interactions is the synthetic lethal relationship between PTEN deficiency and BRG1/SMARCA4 (Fig. 4). PTEN deletion in mouse and human PCa stabilizes BRG1 protein through the AKT/GSK3B/FBXW7 axis, leading to a BRG1-dependent chromatin state that supports oncogenic transcription and growth. Pharmacological targeting of BRG1 selectively suppresses PTEN-deficient tumors both in vitro and in vivo (64), establishing the SWI/SNF ATPase activity as an actionable vulnerability in PTEN-deficient PCa.

A second important context-dependent interaction of PTEN involves CHD1. In PTEN-intact PCa, CHD1 functions to restrain growth and AR occupancy, and defines a molecularly distinct disease subtype compared to PTEN-null PCa, in which CHD1 is essential for growth and survival (70, 71). Mechanistically, PTEN loss stabilizes CHD1 protein again through the AKT/GSK3B axis avoiding proteasomal degradation by B-TrCP, and in turn CHD1 drives TNF-NF-κB signaling and promotes an immunosuppressive tumor microenvironment that facilitates disease progression (Fig. 4) (72). CHD1 undergoes recurrent deletion with recent estimates reporting near 10% loss in primary PCa, whereas higher frequencies occur in advanced stages, in African American patients, and in tumors harboring SPOP loss and SPOP/PTEN loss (73-76). Clinically, CHD1 loss is associated with PSA recurrence and adverse survival outcomes (74, 75). CHD1 loss is associated with a failure to organize chromatin properly at sites of AR-driven transcription, contributing to the genomic instability, altered histone mark distributions and transcriptional reprogramming in CRPC (77-80).

A third synthetic PTEN interaction involves ARID1A, the defining subunit of cBAF complexes (Fig. 2). In Pb-Cre;Pten*fl/fl* prostate mouse models, concurrent Arid1a loss dramatically accelerates tumor progression, shortens survival, and increases metastatic incidence relative to Pten loss alone (81). Integrated cistrome-transcriptome analyses revealed that loss of ARID1A (and the cBAF complex) enhanced AP-1/cFos transcriptional programs leading to altered stromal and differentiation states, implicating the SWI/SNF BAF complex composition as a key regulator downstream of PTEN functions (Fig. 4) (81, 82). These synthetic dependencies suggest that the genomic context of ACR dysregulation, rather than ACR status alone, should inform therapeutic targeting strategies.

### Early therapy-resistant prostate cancer: AR-dependent adaptation under androgen deprivation

As tumors adapt to ARSI, AR remains a central transcriptional driver but operates under attenuated ligand concentrations, associated with a reconfigured enhancer selection and SWI/SNF subunit selectivity. SWI/SNF complexes maintain accessibility at reprogrammed AR enhancers to sustain AR signaling even under these low-androgen conditions. This is mirrored by increased dependency on p300/CBP and BRD4 as co-activators, which may itself represent an early resistance mechanism (83), and justify targeting these proteins therapeutically (84). Similarly, the SWI/SNF ncBAF complex subunit BRD9 is also a critical regulator of AR signaling, whereby its inhibition selectively impairs AR-driven transcription and PCa tumor growth (85). The selective requirement for BRD9/ncBAF suggests non-redundant contributions of this SWI/SNF complex to the AR transcriptional program, indicating multiple BAF complexes may distort AR signaling. The upregulation of additional bromodomain proteins (eg, BRD2 and BRD4) also impact transcriptional events through other mechanisms. BRD2 preferentially associates with acetylated H2A.Z (H2A.Zac) at the +1 nucleosome where its occupancy facilitates RNA Polymerase II promoter escape and productive elongation (86, 87). Given the INO80/SWR antagonism to govern H2A.Zac (88), there is the possibility that ARSI-recurrent tumors have adapted by intensifying dependency on INO80/SWR-controlled +1 nucleosome H2A.Z architecture as a mechanism of AR-bypass transcriptional amplification.

Representative therapy-resistant PCa states arise following RB1/TP53 disruption. Canonically, RB1 enforces G_1_/S checkpoint by repressing E2F-driven transcription through chromatin-based mechanisms that include physical and functional cooperation with SWI/SNF family members including BRG1/SMARCA4-containing complexes (89). In addition, RB1 participates in the response to DNA double-strand breaks, again in conjunction with BRG1, linking cell cycle checkpoint control to repair-associated chromatin remodeling (90, 91). There is also evidence that TP53 interacts with ACRs to mediate transcriptional stress responses, including at damage-response loci and lineage-determining genes. Specifically, TP53 recruits SWI/SNF components to target loci and also recruits INO80/SWR to sites of DNA damage in response to genotoxic stress, underscoring roles for ACRs in chromatin surveillance (92-94).

Genetic targeting of Rb1/p53 in vivo reveals their gatekeeping roles to prevent lineage plasticity and enzalutamide resistance (95, 96), establishing a direct mechanistic connection between tumor suppressor loss, transcriptional reprogramming, and endocrine therapy failure (97). Furthermore, in the context of Rb1/p53 loss, manipulation of Ezh2 reveals Arid1A as an active master regulator in tumors that retain AR expression, suggesting altered SWI/SNF activity is a feature of intermediate lineage plasticity states rather than terminal differentiation to a specific feature such as neuroendocrine differentiation (98). This supports the concept that the Rb1/p53 axis functions to regulate the chromatin landscape broadly in part by cooperation with ACRs, which is distorted under ARSI therapy pressure (96, 99, 100).

### AR-low and AR-indifferent therapy-resistant prostate cancer

During progression to AR-indifference, SWI/SNF transitions from being an AR co-activator to a transcription factor–agnostic chromatin scaffold that enables alternative oncogenic drivers across non-ARE enhancer networks. The glucocorticoid receptor (GR), another classic steroidal nuclear receptor, can function as one of these compensatory transcription factors by substituting for AR at subsets of regulatory sites, and consequently sustain growth under ARSI (101). GR activation is induced by lineage plasticity drivers such as ONECUT2 that suppress the androgen axis. ONECUT2 recruits SMARCA5, suggesting the chromatin accessibility requirements for GR binding overlap with those for AR, placing ACRs, and specifically ISWI, in the position of enabling both canonical and compensatory nuclear receptor programs at a shared set of enhancer loci (102). Recent work has identified co-activating functions between AR and GR, whereby AR expands GR accessibility owing to assisted loading that involves roles for SMARCA4 (103).

ISWI complexes also acquire prominence in AR-indifferent states as facilitators of DNA damage response and chemotherapy tolerance. USP3-mediated stabilization of SMARCA5 enhances homologous recombination capacity and promotes docetaxel resistance, placing ISWI within the stress-adaptive chromatin circuitry that supports AR-indifferent survival (47).This finding is consistent with the broader role of SMARCA5-containing complexes in replication fork stability and chromatin re-establishment following genotoxic insult.

### Neuroendocrine prostate cancer and double-negative prostate cancer

ACRs remain functionally required in the AR-null state but are redirected toward non-AR transcriptional circuits. Neuron-specific BAF subunits enriched in postmitotic neurons, BAF45B/DPF1 and BAF53B/ACTL6B (104), are highly expressed in NEPC, suggesting a subunit-switch paradigm within SWI/SNF that recapitulates the developmental neuronal BAF complex (44). Proteomics demonstrate that SWI/SNF complexes containing SMARCC1 (BAF155) disengage from luminal regulatory factors and instead interact with neural lineage regulators. NEPC-associated SMARCC1 interactions with BAF53B and BAF45B include the neural lineage regulator NKX2.1, neuronal structural components, and members of the NuRD complex MTA1 and CHD4 (44). Specifically, the SWI/SNF complex itself is not lost, but its partnership landscape and regulatory elements it engages are transformed. SMARCA4 actively promotes lineage plasticity and enzalutamide resistance through regulation of the transcription factor, prospero homeobox 1, PROX1 (105), providing a mechanistic link between SWI/SNF ATPase activity, subsequent histone modification state, and the lineage-specific transcriptional programs that define the AR-null phenotype. These epigenomic events may be facilitated by AR-independent pioneers. For example, FOXA2 drives opening of NEPC-associated regulatory elements, developmental super-enhancer hubs, and activation of gene programs with NKX2-1, while luminal enhancers collapse, yielding the therapy resistance and metastatic competence that characterize NEPC (106).

The pioneer factor, HOXB13 displays a particularly instructive pattern of ACR partner selection across disease states. In luminal AR-driven PCa, HOXB13 binds with SMARCC1/BAF155 at H3K27ac-marked active enhancers to sustain luminal identity (44). However, in enzalutamide-resistant contexts, HOXB13 interactions with SMARCC1 and SMARCA4 are reduced, whereas its interactions with the tissue-specific SMARCD2/BAF60B partner to SMARCD2 (BAF60B) SWI/SNF subunit is maintained in both adeno/AR-dependent and AR-independent PCa models, including AR-negative PC3 cells, to promote chromatin accessibility (107, 108). SMARCD subunits occupy mutually exclusive positions within SWI/SNF complexes (Fig. 2) and actually display context-dependent relationships with AR signaling, functioning as co-activators or co-repressors (109).

Finally, the double-negative PCa (DNPC) subtype, defined by loss of both AR and neuroendocrine markers, yields a spectrum of molecular heterogeneity. These aggressive phenotypes can be enriched for WNT-dependent as well as WNT-independent, stem cell like chromatin accessibility programs (110). Again, SMARCA2/4 have emerged as central to sustaining TCF7L2-centered chromatin programs in these PCa-WNT subtypes and promoting in vivo tumor growth (111). This further emphasizes that SWI/SNF-enabled enhancer dependencies are sustained throughout the spectrum of PCa progression and through non-AR transcription factor partners, likely a dependency in DNPC tumors as the majority harbor biallelic PTEN loss (112). The genomic and epigenomic landscape of DNPC is characterized by widespread chromatin remodeling relative to both AR-positive ARSI-independent and NEPC yet appear to share active TFs on accessible chromatin (112) underscoring that this ARSI-resistant subtype represents a genuine chromatin reprogramming event rather than a simple loss of AR activity. Nucleosome remodeler expression of CHD7 appears to be selectively enriched for androgen-independence, as both DNPC and NEPC express high levels of CHD7, associated with copy number gain and enhancer hypomethylation, respectively (112). This represents an opportunity to interrogate CHD7 activity in AR-negative models.

## Therapeutic opportunities

### Targeting SWI/SNF ATPases and other subunits

Of the 4 major ACR subfamilies, the SWI/SNF family represents the only family in clinical stage for therapeutic targeting. Although SMARCA4 mutation is relatively uncommon in PCa compared to other cancer types (113, 114), functional SMARCA4 dependency, especially in the context of PTEN loss (Fig. 4), creates a de facto vulnerability that is exploitable with degrader molecules in a genomically defined patient subset. Likewise, SMARCA4 promotion of lineage plasticity and enzalutamide resistance (105) also provides a further rationale for targeting SWI/SNF ATPase activity in the enzalutamide-resistant setting. The dual SMARCA2/4 degrader AU-15330 suppresses growth in enhancer-addicted PCa (64). A dual degrader (FHD-286) tested in Phase I (NCT04891757) for advanced myeloid malignancies reported no objective responses (115). A complementary orally bioavailable degrader of SMARCA2, SMARCA4, and PBRM1 (AU-24118) has demonstrated efficacy in preclinical ARSI-resistant models but also highlighted acquired resistance mechanisms that bypass chromatin opening requirement (116). Likewise, a PROTAC-mediated SMARCA2 degrader tested as an oral compound (ABCI2) demonstrated high selectivity and decreased tumor growth in mouse lung xenografts. While novel degraders remain actively in development (117), one SMARCA2-selective inhibitor (FHD-909/LY4050784) is in Phase I (NCT06561685) for non-small cell lung cancer although other cancers, including PCa, with SMARCA4 alterations are eligible as future indications. More recently, SWI/SNF dependency has been demonstrated in preclinical models of AR-indifferent PCa models (111), suggesting that SWI/SNF targeting may be extended beyond AR-positive disease.

BRD9 degraders have shown preclinical activity across multiple cancer types and have entered Phase I (NCT04965753) in synovial sarcoma or SMARCB1 loss. Clinical evaluation of BRD9 PROTAC degrader (FHD-609) targeting the ncBAF complex has demonstrated proof-of-mechanism by achieving extensive degradation in tumor tissue with downregulation of proliferative gene expression programs (118). However, the single-agent efficacy was minimal and demonstrated cardiac toxicity, with some patients presenting stable response and only one presenting partial response, likely a consequence of enhancing ncBAF remodeling capacity as recently reported (119). In PCa, BRD9 inhibition represents an opportunity to disrupt the AR axis, as BRD9 degradation impairs AR-driven transcription in vitro and reduced tumor growth and development in vivo following BRD9-inducible knockdown (85). The selectivity of BRD9 for the ncBAF complex is also attractive, as this complex lacks SMARCB1 and ARID subunits and therefore has a distinct genomic targeting profile from cBAF and pBAF (Fig. 2). As a result, BRD9 targeting may perturb a specific subset of AR-regulated enhancers without the broader chromatin consequences of dual ATPase degradation, potentially yielding a higher therapeutic index, a hypothesis that warrants formal testing.

### Targeting ISWI and INO80/SWR

Within the ISWI family, the BAZ2A-containing NoRC complex has been most extensively characterized as a PCa-relevant therapeutic target. BAZ2A (TIP5) is overexpressed in PCa and its overexpression predicts disease recurrence, establishing it as a clinically relevant biomarker and potential target (120). Mechanistically, BAZ2A promotes PCa stem cell properties (121), and BAZ2A association with TOP2A and KDM1A represses genes implicated in PCa suppression (122). This series of studies are impactful as they establish BAZ2A as an oncogenic ISWI complex subunit that is distinct from SWI/SNF, acting on heterochromatin organization and enhancer repression rather than chromatin opening. The BAZ2A bromodomain is a tractable small molecule target, and early inhibitor development has indicated selectivity, providing a direct path toward therapeutic exploitation.

By contrast, SMARCA5 presents a more complex biological and therapeutic picture. There is evidence for this ATPase being dosage-sensitive, in part because circSMARCA5 and SMARCA5 are co-expressed from the same locus and produce functionally opposing outputs. In hematopoietic malignancies and breast cancer, SMARCA5 is overexpressed and supports proliferative undifferentiated states, and its loss impairs tumor cell proliferation. In PCa, by contrast, SMARCA5 dysregulation operates through altered nucleosome phasing at lineage-specific regulatory elements rather than bulk proliferative support, and the direction of change is ancestry-associated, underscoring that SMARCA5 function cannot be generalized across cancer types (47, 50).

Finally, the INO80 complex presents a different therapeutic angle in PCa. Its role in R-loop resolution and replication fork stability makes it a determinant of replication stress tolerance that is required to maintain cancer cell proliferation and viability (54). INO80 inhibition may therefore sensitize PCa cells to agents that exacerbate replication stress, including PARP inhibitors, topoisomerase inhibitors, and potentially AR-driven transcription amplification itself, which generates transcription-replication conflicts at AR target loci (123-125).

## Conclusion

ACRs demonstrate both distinct and integrated roles to control epigenomic organization that are PCa-driving and provide routes to therapeutic resistance. Collectively, ACRs functions support a model whereby chromatin remodeler rewiring is structurally integrated into the regulatory circuitry controlling the transition from canonical AR-dependent adenocarcinoma to AR-independent, and ultimately to ARSI-resistant PCa. In AR-driven adenocarcinoma, SWI/SNF complexes cooperate with the AR transcriptional network to stabilize enhancer landscapes that sustain luminal identity and AR-dependent proliferation. In advanced PCa states, AR-dependent programs collapse and there is clear evidence that SWI/SNF, ISWI, and INO80/SWR family members are all redirected to non-AR circuits governing stem-like, neuronal, and neuroendocrine and other lineage states. Lineage plasticity drivers appear to be at the interface of these redirections and capable of remodeling super-enhancers and interacting further with ACR complexes to promote androgen indifferent cell states (102). This shift appears instructive rather than passive, reflecting the active reprogramming of chromatin architecture as a determinant of disease progression and therapy resistance.

## Data Availability

No new data were created or analyzed in this study. Data sharing is not applicable to this article.

## Abbreviations

ACR: ATP-dependent chromatin remodeler
AR: androgen receptor
ARSI: androgen receptor signaling inhibitor
BAF: BRG/BRM-associated factor
CHD: Chromodomain Helicase DNA-binding
ERα: estrogen receptor alpha
NURF: Nucleosome Remodeling Factor
PCa: prostate cancer
